# Society Meetings

**Published:** 1900-03

**Authors:** 


### SOCIETY MEETINGS.

THE Medical Union of Buffalo, a private medical club, held its eighteenth annual banquet at the Genesee Hotel, the fourth Tuesday in January. Toasts were responded to as follows: By Dr. Roswell Park, on the State Laboratory; by Dr. J. C. Thompson, on the Medical Union; by Surgeon-Maj. A. H. Briggs, of the 65th Regiment, on The army; by Dr. Frederick Preiss, on The ladies, and by Dr. S. S. Green, on The new member.

The banquet committee was composed of Maj. A. H. Briggs, Dr. A. A. Hubbell, Dr. J. C. Thompson and Dr. Frederick Preiss.

The officers for the ensuing year are: Dr. J. C. Thompson, president; Dr. Herman Mynter, vice-president; Dr. Frederick Preiss secretary.

---

THE American Medico-Psychological Association will hold its annual meeting in Richmond, Va., May 8, 9, 10, 11, 1900—not May 1, 2, 3, 4, as heretofore announced. The change in date is made to enable members to attend the congress of American Physicians and Surgeons in Washington, May 1-4.

---

THE Buffalo Academy of Medicine held meetings during the month of February, 1900, as follows:

*Section on Surgery.* — Wednesday evening February 7th. Program: Remarks on tubercular peritonitis, Gastrostomy, Acute appendicitis, Cure of malignant diseases by surgical intervention, Frederick S. Dennis.

*Section on Ophthalmology, Otology and Laryngology.*—Monday evening, February 12th. Program: Some improvement in operating $x$-ray apparatus, Elmer Starr; Radical operation for caries of middle ear, with exhibition of cases, George F. Cott; Report of a case of retropharyngeal abscess, Irving M. Snow.

*Section on Medicine.*—Tuesday evening, February 13th. Program: Dress reform, Matthew D. Mann; discussion by Hopkins, Bartow and Stockton.

*Section on Pathology.*—Tuesday evening, February 20. Program: Diseases of the skin, illustrated by stereopticon, Grover W. Wende; New method of analysis of stomach contents, A. L. Benedict.

*Section on Obstetrics.*—Tuesday evening, February 27th. Program: Indications for and selection of operation for uterine myomata, Edward J. Ill, of Newark N. J.

---

AN International Congress of Medical Electrology and Radiology will take place in Paris from the 27th of July to the 1st of August, 1900. All inquiries for further information should be forwarded to Professor E. Doumer, general secretary, 57 Rue Nicolas-Leblanc, Lille. Membership subscriptions are to be sent to Dr. Moutier, 11 Rue de Miromesnil, Paris.

---

THE regular monthly meeting of the Niagara Falls Academy of Medicine was held at the office of Dr. C. T. Leo-Wolf, Gluck Building, Monday, February 5, 1900, 8.30 p. m. Dr. Guillemont read a paper on Neurasthenia. The discussion was opened by Dr. McBlain.

